# A Molecular Method for the Identification of Honey Bee Subspecies Used by Beekeepers in Russia

**DOI:** 10.3390/insects9010010

**Published:** 2018-01-27

**Authors:** Mikhail Y. Syromyatnikov, Anatoly V. Borodachev, Anastasia V. Kokina, Vasily N. Popov

**Affiliations:** 1 Department of Genetics, Cytology and Bioengineering, Voronezh State University, Voronezh 394018, Russia; nastenka.kokina@mail.ru (A.V.K.); pvn@bio.vsu.ru (V.N.P.); 2 Department of Bee Selection, Federal Beekeeping Research Center, Rybnoye 391110, Russia; rybnoebee@mail.ru

**Keywords:** *Apis mellifera*, subspecies, DNA barcoding, SNP, PCR-RFLP

## Abstract

*Apis mellifera* L. includes several recognized subspecies that differ in their biological properties and agricultural characteristics. Distinguishing between honey bee subspecies is complicated. We analyzed the Folmer region of the *COX1* gene in honey bee subspecies cultivated at bee farms in Russia and identified subspecies-specific SNPs. DNA analysis revealed two clearly distinct haplogroups in *A. mellifera* *mellifera*. The first one was characterized by multiple cytosine-thymine (thymine–cytosine) transitions, one adenine-guanine substitution, and one thymine–adenine substitution. The nucleotide sequence of the second haplogroup coincided with sequences from other subspecies, except the unique C/A SNP at position 421 of the 658-bp Folmer region. *A. mellifera* *carnica* and *A. mellifera* *carpatica* could be distinguished from *A. mellifera* *mellifera* and *A. mellifera* *caucasica* by the presence of the A/G SNP at position 99 of the 658-bp Folmer region. The G/A SNP at position 448 was typical for *A. mellifera* *carnica*. *A. mellifera* *caucasica* *COX1* sequence lacked all the above-mentioned sites. We developed a procedure for rapid identification of honey bee subspecies by PCR with restriction fragment length polymorphism (RFLP) using mutagenic primers. The developed molecular method for honey bee subspecies identification is fast and inexpensive.

## 1. Introduction

The honey bee (*Apis mellifera* L.) is a major producer of honey. Additionally, honey bees are important pollinators of entomophilic crops [1]. Several bee breeds (subspecies) are recommended for breeding within the territory of the Russian Federation. The Central Russian (*Apis mellifera mellifera*), Carpathian (*Apis mellifera carpathica*), Grey Mountain Caucasian (*Apis mellifera caucasica*), Bashkir breeds, and some breed lines of Central Russian (*Apis mellifera mellifera*) were included to the State register of Russian bees approved for breeding (http://reestr.gossort.com/reestr/animal/710). One of the important areas of bee breeding is the preservation of bee subspecies genotypes diversity, which can be used as a starting material for breeding and crossbreeding. Breed purity is an important factor in these processes. In Russia, there are more than 20 breeding bee farms, and there are *Apis mellifera* populations from the Primorye region. It is believed that these bees are more resilient to parasitic mites [2,3]. All of the above factors determine the importance of correctly identifying honey bee subspecies in Russia.

The breeding of *A. mellifera carpatica* in the Federal state unitary enterprise at the bee breeding farm “Maikop,” Republic of Adygea, Russia, began in 1986 with the delivery of initial breeding material from the Mukachevo (Western Ukraine) bee farm. Since 2002, egg laying queens of *A. mellifera carnica* were brought by this farm from the Austrian Institute of Apiculture, Lunz am See (AIA), and the Institute of Kirchhain, Germany. Breeding of the *A. mellifera caucasica* began in the “Krasnopolyanskaya State Experimental Station of beekeeping” in 1963. The original biomaterial (Mingrelian, Abkhazia, and Kartli populations of gray mountain Caucasian bees breed) was imported from the mountainous apiaries of Georgia. The breeding group was improved with gray mountain Caucasian bees, which differ in comparison with the introduced populations in terms of increased hardiness, egg laying, and productivity. It was formed after production testing and the selection of the most productive and typical exterior characteristics of bee families. *A. mellifera mellifera* were selected in the Republic of Bashkortostan (Ufa district) from their natural habitat. Distinguishing between honey bee subspecies is complicated and requires specific knowledge. It is generally based on bee morphometric characteristics, such as head width in frontal view (including eyes), length of the left antennal scape, and length of the left hind tibia [4]. Wing morphometrics is also commonly used [5,6,7,8,9]. Forewing angles were used to distinguish between bee groups [10,11]. Additionally, a fully automated image analysis system has been determined to be an efficient tool for insect identification [12]. The major disadvantages of all morphology-based methods are a lack of qualified specialists and difficulties in preparing specimens for analysis.

An alternative approach to distinguishing between honey bee subspecies is the use of genetic markers, e.g., analysis of nuclear DNA microsatellite loci [13,14]. However, the widespread application of this method is prohibited by the necessity of expensive equipment (capillary electrophoresis system), a high cost of analysis, and potential misinterpretation of results due to a possible overlapping of loci in different bee subspecies [15].

At the moment, the most widely used genetic method for distinguishing between bee subspecies is the analysis of the mitochondrial DNA fragment located between the genes for cytochrome oxidase c subunits 1 and 2 (*COX1* and *COX2*) [16,17,18]. This fragment is AT-enriched and significantly differs in its length and nucleotide composition between honey bee populations [19,20]. In most cases, the amplified fragment that is located between *COX1* and *COX2* is treated with *Dra*I restriction endonuclease that recognizes the TTTAAA sequence [16,17], and the products of digestion are analyzed. The AT-enriched region appears to contain valuable information, especially at the population level [21].

Nucleotide polymorphism of nuclear DNA has also been used for species identification. Thus, it was found that a fragment of the honey bee sex determination gene exhibits high interspecific variability [22]. The major drawback of nuclear DNA analysis is the presence of two alleles of the same gene, which requires their preliminary separation by molecular cloning.

Molecular genetic analysis of the subspecies of honey bees are also needed to assess the introgression of alien populations. The analysis of mitochondrial DNA and genome-wide SNP has shown that even protected populations of *A. mellifera mellifera* carried alien alleles [23]. In addition, a comparison of resolution for microsatellite analysis and SNP analysis based on high-throughput sequencing has shown that, for native subspecies of the European *A. m. mellifera* and the commercial *A. m. mellifera*, SNPs are more accurate and powerful than microsatellites for identification of *A. m. mellifera* [24]. Moreover, it has been shown that the single nucleotide polymorphisms of *Apis mellifera* can be strongly associated with one or more environmental variables [25].

DNA barcoding is widely used to identify organisms. It includes the amplification and sequencing of the mitochondrial DNA of the *COX1* gene. The obtained nucleotide sequence is compared to the sequences deposited in the databases, such as Bold system and GenBank [26,27,28,29]. This DNA fragment is highly conserved within the same taxon but greatly varies between different taxa. Despite the wide acceptance of DNA barcoding, its use for the identification of honey bee subspecies has been significantly limited [30].

The goal of this study was to analyze the Folmer region of the *COX1* gene in honey bee subspecies cultivated at bee husbandry farms in Russia to identify subspecies-specific SNPs and to develop a method for the rapid molecular genetic identification of Russian honey bee subspecies.

## 2. Materials and Methods

### 2.1. Bees Collection and Morphological Analysis

*A. m. carpathica* and *A. m. carnica* breeds were collected from apiaries of the Maykopskoe Federal Bee Husbandry Facility (Republic of Adygea, Russia), *A. m. caucasica* from the Krasnopolyanskaya Experimental Bee Husbandry Station (Krasnodar region, Russia), and *A. m. mellifera* from the Republic of Bashkortostan, Russia. Proboscis length, third tergit width, and cubital and tarsal indexes are the main features that characterize the breed of bees in Russia. The analysis of these characteristics was performed according to the method of V.V. Alpatov (1948). Morphometric parameters are shown in Table 1.

### 2.2. DNA Barcoding

DNA was isolated from bee legs with a ZR Tissue & Insect DNA MicroPrep kit (Zymo Research, Irvine, CA, USA). Voucher specimens were stored at the Department of Ecology and Systematics of Invertebrates, Voronezh State University. PCR was performed with an Eppendorf MasterCycler Personal cycler. Each PCR reaction mixture contained 2.5 µL of 10× reaction buffer (Evrogen, Moscow, Russia), 1 µL of 10 mM dNTPs, 1 µL of 10 µM forward primer, 1 µL of 10 µM reverse primer, 3 µL of 25 mM Mg^2+^, 1 µg of template DNA, 2.5 units of thermostable Taq DNA polymerase (Evrogen, Moscow, Russia), and deionized water (up to 25 µL). PCR regime included initial denaturation at 94 °C for 3 min; 35 cycles of denaturation at 94 °C for 30 s, annealing at 51 °C for 30 s, elongation at 72 °C for 45 s; and final elongation at 72 °C for 10 min. Primers used for DNA barcoding were: LepF1, LepR1 [31,32]. Additionally, we used AmCarp-f and AmCar-r primers developed by us (Table 2).

Cytochrome b gene amplification was performed using CYTB-f and CYTB-r primers (Table 2) undo temperature cycle: 94 °C for 3 min; 35 cycles of denaturation at 94 °C for 30 s, annealing at 51 °C for 30 s, elongation at 72 °C for 40 s; and final elongation at 72 °C for 10 min.

PCR products were separated by electrophoresis in 2% agarose gel, stained with ethidium bromide, and visualized with a TCP-20LM transilluminator at 312 nm. The size of PCR products was determined using 100+ DNA length standards (Evrogen, Moscow, Russia).

PCR products were purified from an agarose gel with a Cleanup Standard kit (Evrogen, Moscow, Russia) and sequenced with an Applied Biosystems 3500 genetic analyzer using the BigDye Terminator v3.1 Cycle Sequencing Kit. DNA barcoding primers (LepF1, LepR1, ApMel-r CAGCTAATACAGGTAATGA, ApMel-f AGATATTGGGATCTTGTA, CYTB-f TATGTACTACCATGAGGACAAATATC and CYTB-r ATTACACCTCCTAATTTATTAGGAAT) were used for sequencing. Sequence alignment was performed with the Clustal Omega tool (The European Bioinformatics Institute, Hinxton, UK, http://www.ebi.ac.uk/Tools/msa/clustalo). Sequences were translated into amino acid sequences with the EMBOSS Transeq program (The European Bioinformatics Institute, Hinxton, UK, http://www.ebi.ac.uk/Tools/st/emboss_transeq/) to verify that they contained no stop codons or gaps.

A neighbor-joining tree was constructed using the Kimura 2-parameter method [33] in MEGA6 [34]. The tree is drawn to scale, with branch lengths in the same units as those of the evolutionary distances used to infer the phylogenetic tree. The percentage of replicate trees in which the associated taxa clustered together in the bootstrap test (500 replicates with pairwise deletion of gaps/missing data and inclusion of all substitutions (transitions and transversions)) are shown next to the branches. *Apis cerena* was chosen as the outgroup.

### 2.3. PCR-RFLP

Restriction endonucleases for species differentiation were selected using theoretical diagrams of DNA digestion by enzymes available from Sibenzyme (Sibenzyme, Novosibirsk, Russia http://www.sibenzyme.com/products/restrictases). PCR products (10 µL) were digested in a reaction mixture containing 1.5 µL of 10X reaction buffer and 10 U of Alu I, Hinf I, HspA I, and Msp I restriction endonuclease (Sibenzyme, Novosibirsk, Russia) in a total volume of 15 µL. The mixture was incubated for 2 h at 37 °С, and the enzyme was then inactivated at 75 °С for 15 min. The digestion products were visualized after fractionation by electrophoresis in 3% agarose gel.

## 3. Results

### 3.1. DNA Barcoding

Bees were pre-identified according to their morphology (see Materials and Methods). Twenty to 30 bees for each subspecies were initially studied (2–3 bees from each bee colony). We performed classical DNA barcoding (amplification of the Folmer region) using the LepF1/LepR1 primers. However, amplification efficiency with this primer pair was very low.

We were still able to amplify a number of honey bee DNA sequences using the LepF1/LepR1 primers pair. We also developed an alternative pair of primers (AmCarp-f and AmCar-r) that yielded a product of 543 bp, and we used the AmCar-f /LepR1 primer combination to obtain a longer fragment of 663 bp. We amplified a total of 51 nucleotide sequences that were aligned and deposited in the GenBank under the following accession numbers: *A. mellifera mellifera* KY271928.1–KY271939.1, *A. mellifera carnica* KY271901.1–KY271916.1, *A. mellifera carpatica* KY271917.1–KY271927.1, and *A. mellifera caucasica* KY271890.1–KY271900.1 (see nucleotide sequences in Supplementary materials). Using the MEGA6 software, we searched for single-nucleotide polymorphisms (SNPs) characteristic of each of the analyzed subspecies and estimated the intrabreed nucleotide variability of the *COX1* gene. We found that the highest intrabreed variability of the *COX1* gene was in *A. mellifera mellifera* and then analyzed nucleotide substitutions typical of this subspecies.

DNA analysis revealed two clearly distinct haplogroups in *A. mellifera mellifera*. The first one was characterized by multiple cytosine-thymine (thymine–cytosine) transitions, an adenine-guanine substitution, and a thymine–adenine substitution. To develop a method for rapid subspecies identification, we used the C/T SNP at position 421 of the 658 bp Folmer region. The nucleotide sequence of the second haplogroup coincided with sequences from other subspecies, except the unique C/A SNP at position 421 of the 658 bp Folmer. SNPs typical for the second haplogroup of the mid-Russian breed were found in 25% of bees. 

*A. mellifera carnica* and *A. mellifera carpatica* could be distinguished from *A. mellifera mellifera* and *A. mellifera caucasica* by the presence of the A/G SNP at position 99 of the 658 bp Folmer region. The G/A SNP at position 448 was typical for *A. mellifera carnica*; however, in two specimens, this SNP was absent, i.e., their *COX1* sequence were identical to the *COX1* sequence of *A. mellifera carpatica*. None of the above-mentioned SNPs was found in *A. mellifera caucasica*.

**Figure 1:**
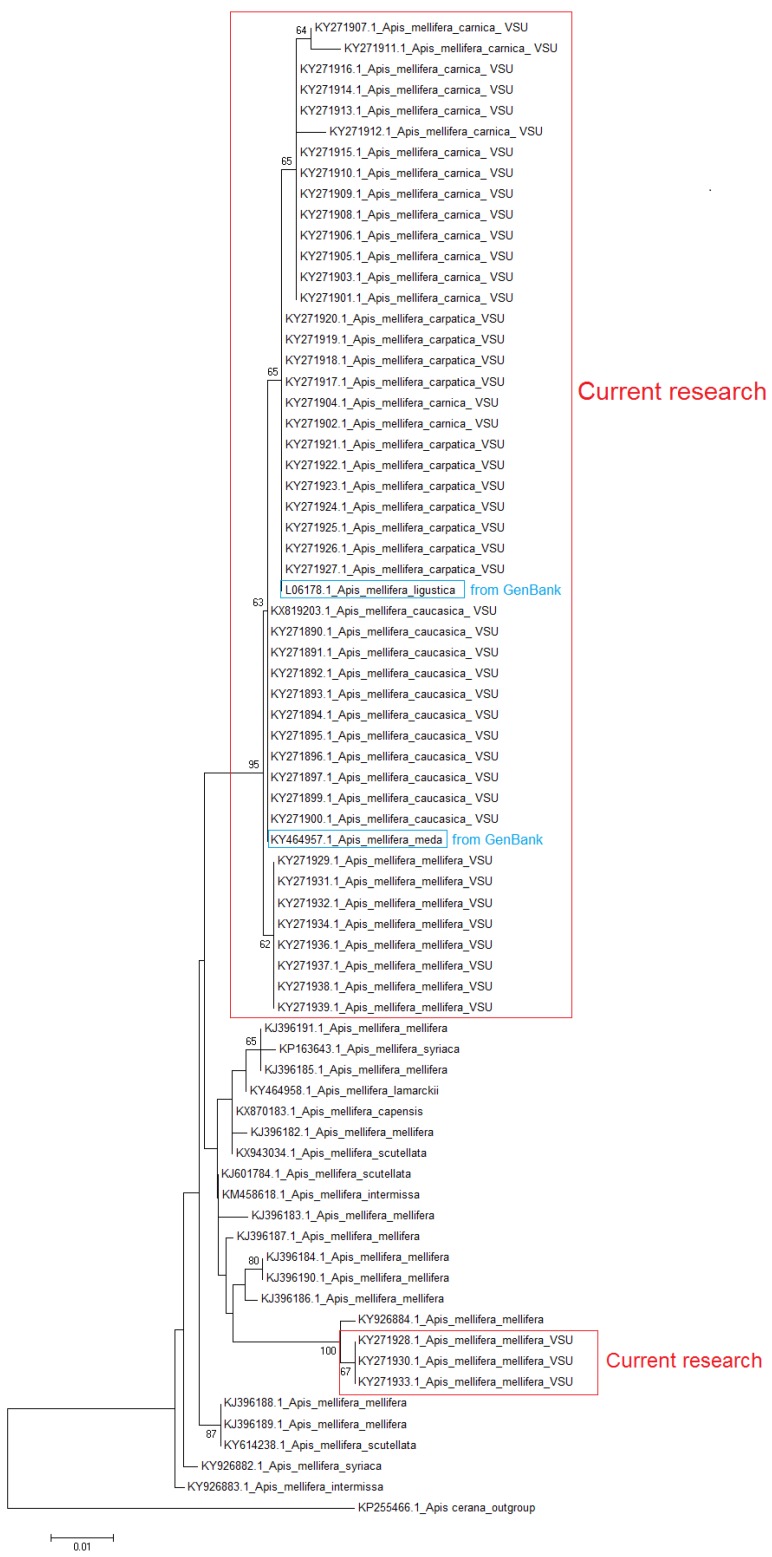
Neighbor joining analysis of *COX1* gene sequences from the *Apis mellifera* subspecies.

Finally, we constructed a phylogenetic tree that reflects the genetic distances between *Apis mellifera* subspecies using the Kimura 2-parameter algorithm and the *COX1* gene sequences of *Apis mellifera* subspecies we obtained as well as all *Apis mellifera* subspecies sequences available in the GenBank database (Figure 1).

### 3.2. Development PCR-RFLP

Next, we developed a procedure for the rapid identification of honey bee subspecies using restriction fragment length polymorphism (RFLP). Preliminary in silico screening revealed no suitable combination of restriction enzymes that would allow us to distinguish between honey bee subspecies using the classical PCR-RFLP method. Therefore, we designed a set of primers to cytochome oxidase subunit 1 gene of mitochondrial DNA for PCR with mutagenic primers (Table 3), so that the resulting PCR products were 120–160 bp in length to ensure their separation in agarose gel.

The developed primer pairs yielded PCR products whose length varied from 138 to 150 bp. The mutagenic primers contained single or double nucleotide substitutions at the 3′ ends that, in combination with the identified characteristic SNPs, resulted in the formation of unique restriction endonuclease recognition sites in the PCR products (see Table 4 for restriction endonucleases and resulting digestion fragments).

**Figure 2:**
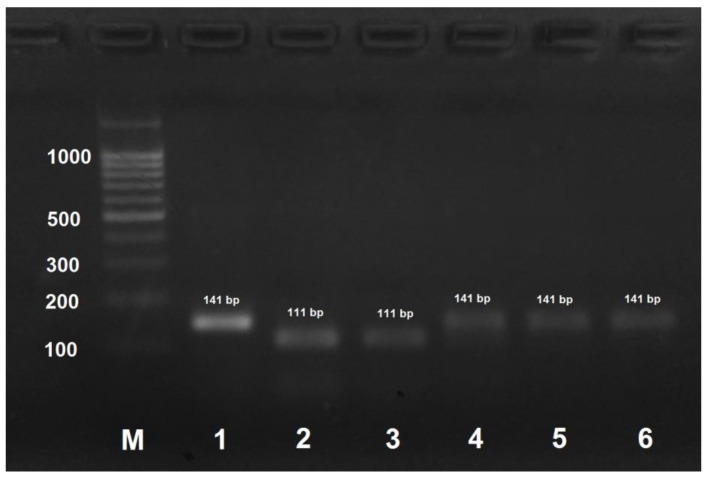
PCR products obtained by the amplification of honey bee DNA with AmCarp-f/AmCarp-r primers and treated with the HspAI restriction enzyme. M: DNA ladder, bp; 1: PCR product before treatment with restrictase; 2: *A. mellifera carpatica*; 3: *A. mellifera carnica*; 4: *A. mellifera mellifera* haplotype 1; 5: *A. mellifera mellifera* haplotype 2; 6: *A. mellifera caucasica*.

PCR product obtained by the amplification of *A. mellifera carpatica* and *A. mellifera carnica* DNA using the AmCarp-f/AmCarp-r primer pair and treated with HspAI yielded two fragments of 111 and 30 bp (Figure 2). For all the other honeybee subspecies, PCR products obtained by DNA amplification with the same primer pair were not digested with HspAI.

**Figure 3:**
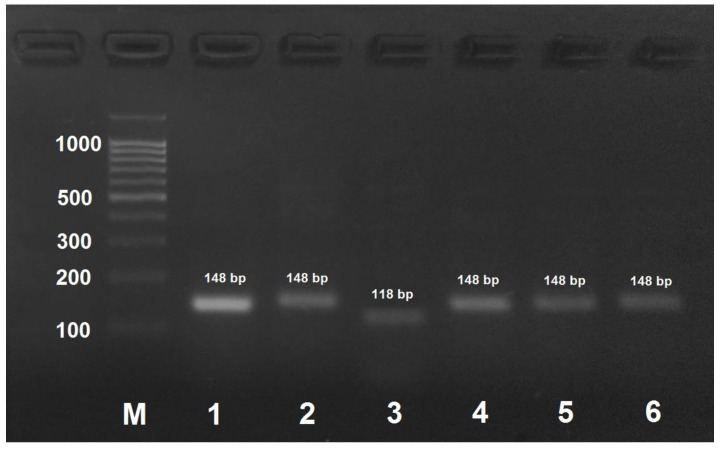
PCR product obtained by the amplification of honey bee DNA with AmCar-f/AmCar-f primers and treated with the MspI restriction enzyme. M: DNA ladder, bp; 1: PCR product before treatment with restrictase; 2: *A. mellifera carpatica*; 3: *A. mellifera carnica*; 4: *A. mellifera mellifera* haplotype 1; 5: *A. mellifera mellifera* haplotype 2; 6: *A. mellifera caucasica*.

*A. mellifera carnica* could be distinguished from other subspecies by DNA amplification with AmCar-f/AmCar-f primers followed by treatment of the PCR product with MspI that results in the formation of two digestion fragments of 118 and 30 bp (Figure 3). PCR products obtained by the amplification of DNA from the other subspecies were not digested with MspI.

However, for 13% of *A. mellifera carnica* specimens, the RFLP pattern was identical to that of *A. mellifera carpatica* due to the identity of the analyzed sequences (see above). Therefore, in some cases, this approach will be insufficient for distinguishing between *A. mellifera carnica* and *A. mellifera carpatica*.

**Figure 4:**
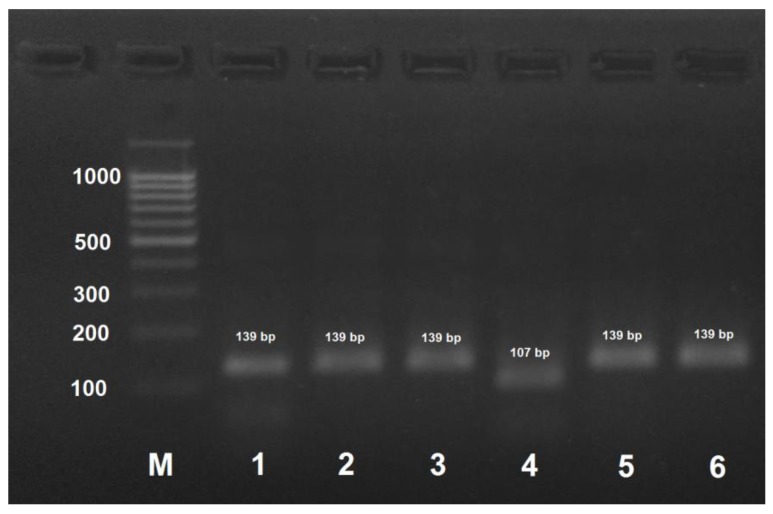
PCR product obtained by the amplification of honey bee DNA with AmEu1-f/AmEu1- primers and treated with the AluI restriction enzyme. M: DNA ladder, bp; 1: PCR product before treatment with restrictase; 2: *A. mellifera carpatica*; 3: *A. mellifera carnica*; 4: *A. mellifera mellifera* haplotype 1; 5: *A. mellifera mellifera* haplotype 2; 6: *A. mellifera caucasica*.

*A. mellifera mellifera* haplotype 1 could be identified by PCR with AmEu1-f/AmEu1-r followed by the treatment of the obtained PCR product with AluI that yields two digestion products of 107 and 32 bp. (Figure 4). PCR products for the other three subspecies and *A. mellifera mellifera* haplotype 2 are not digested with AluI.

**Figure 5:**
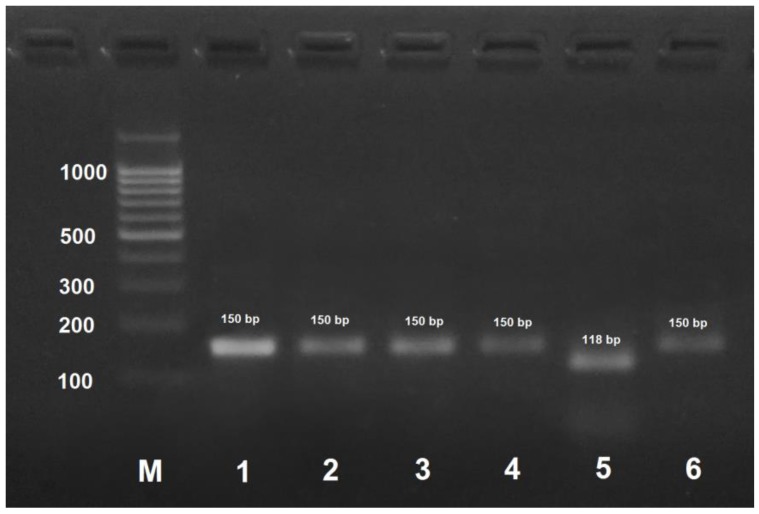
PCR product obtained by the amplification of honey bee DNA with AmEu2-f/AmEu2- primers and treated with the HinfI restriction enzyme. M: DNA ladder, bp; 1: PCR product before treatment with restrictase; 2: *A. mellifera carpatica*; 3: *A. mellifera carnica*; 4: *A. mellifera mellifera* haplotype 1; 5: *A. mellifera mellifera* haplotype 2; 6: *A. mellifera caucasica*.

Similarly, *A. mellifera mellifera* haplotype 2 could be distinguished from the other subspecies and haplotype 1 by PCR with AmEu2-f/AmEu2-r primers with subsequent treatment of the PCR product with HinfI that yielded digestion fragments of 122 and 28 bp (Figure 5). PCR products for the other three subspecies and *A. mellifera mellifera* haplotype 1 were not digested with HinfI.

The *A. mellifera caucasica* sequence lacked all of the above-mentioned sites. This subspecies should be identified by PCR reactions with all the developed primer pairs (Table 2) followed by the digestion of the obtained PCR products with the corresponding restriction endonucleases.

## 4. Discussion

The goal of our study was to develop a procedure for the molecular identification of honey bee subspecies cultivated within the territory of the Russian Federation. We found that these subspecies differ by the nucleotide sequence of the mitochondrial *COX1* gene. We suggested an alternative highly specific direct primer AmpCarp-f that, together with the reverse LepR1 primer, will yield a shorter 663-bp DNA fragment of the *COX1* gene that includes all SNPs required for the honey bee subspecies identification.

Neighbor joining analysis showed that the three Russian breeds of *A. mellifera* subspecies: *A. m. caucasica*, *A. m. carnica*, and *A. m. carpathica,* as well as one of the haplotypes of *A. m. mellifera* are represented by a separate cluster. However, the second haplotype of *A. m. mellifera* is represented as a separate cluster, together with other *A. m. mellifera* subspecies from GenBank. In general, there is a low level of bootstrap value, but it should be taken into account that *Apis mellifera* breeds are not species, but subspecies. Additionally, it should be noted that the identified SNP are stable, and this makes it possible to develop a method for differentiating subspecies of *Apis mellifera*. It is interesting that the DNA sequence of the *Apis mellifera ligustica*, taken from the GeneBank, is in the same cluster in which *A. m. carpathica* and *A. m. carnica* are. *A. m. meda* is in a cluster with *A. m. caucasica*. It should also be noted that the neighbor joining analysis of COX1 sequences is useless for the differentiation of *A. m. scutellata, A. m. mellifera, A. m. syriaca, A. m. intermissa, A. m. capensis*, and *A. m. lamarckii.* Generally, using neighbor joining analysis of COX1 sequences is limited to the identification of honey bee subspecies.

The *COX1* gene is commonly used for species identification (including bees) [35,36]. However, we found that *COX1* sequences also differ in honey bee subspecies and exhibit stable SNPs typical for individual subspecies. DNA barcoding has been used for distinguishing honey bee subspecies in Turkey [37]; the authors demonstrated that the *COX1* sequence differs between different bee subspecies. Estimation of the interspecific variability of the *COX1* gene in honey bees using the MEGA6 tool showed that this parameter is equal to 0.007, which indicates that the analyzed DNA fragment is a variable region. The values for the variability of the *COX1* genes within a breed differed for the breed analyzed, with the highest variability (0.016) in *A. mellifera mellifera*. The variability of the *COX1* gene in *A*. *mellifera carnica* was lower (0.002). In *A. mellifera caucasica* and *A. mellifera carpatica*, the *COX1* gene showed no variability at all. Interestingly, SNPs in the *COX1* sequences of *A. mellifera mellifera* haplotype 1 and *A. mellifera caucasica* were the same as in the sequences deposited in the GenBank, thereby indicating the stability of the mitochondrial genome in bee subspecies despite their geographical separation. The *COX1* gene of *A. mellifera carpatica* has been sequenced by us for the first time.

It should be noted that the disadvantage of mitochondrial DNA studies is the difficulty in tracing paternal honey bee introgression, particularly with African species, such as *A. m. scutellata*. To track the paternal introgression, it is necessary to study nuclear DNA. The historical processes of the paternal nuclear introgression for *A. m. scutellata* in Europe and North America have been shown via nuclear DNA analyses for different bee subspecies [38,39], while mitochondrial DNA did not allow for to detect these processes. However, the introgression of African bees is not a problem in Russia due to the cold climatic conditions, and no evidence of such a process has been found.

In this study, we developed a PCR-RFLP procedure for the rapid identification of honey bee subspecies. The amplified region of cytochome oxidase subunit 1 gene of mitochondrial DNA was 130–150 bp in size; the length of the cleaved-off fragment (if the site for restriction nuclease was present) was determined by the length of the mutagenic primer (~30 nucleotides), which allowed easy visualization of the reaction products in 3% agarose gel (agarose gel is preferred to polyacrylamide gel because of an easy preparation procedure and a low cost of reagents). When the amplified DNA fragment originally contained no restriction site characteristic of a particular SNP, we used primers containing nucleotide substitutions at the 3′ end to introduce restriction nuclease recognition sites into the amplifications products. Despite the absence of complementarity at the 3′–end, mutagenic primers hybridized with the template DNA. It should be noted that the primers alone are not specific for honey bee subspecies, restriction is absolutely necessary. In rare cases, the template DNA was too degraded (e.g., due to the long-term storage of the samples), so that the primers failed to hybridize on it. In this case, PCR was carried out in two steps: the first amplification was performed with the highly specific AmCarp-f/AmCar-r primers that yielded a 572 bp DNA fragment containing all SNPs required for honey bee subspecies identification. The obtained PCR product (1 µL of the PCR reaction mixture) was then used for amplification with the subspecies-specific primers to verify the honey bee breed (subspecies) or, if necessary, with all the primer pairs, and the obtained products were then treated with the corresponding restriction endonucleases. Alternative restriction endonucleases with identical recognition sites could be used in the developed procedure (AluBI instead of AluI; HinP1I, AspLEI, BstHHI, CfoI, GlaI, and Hin6I instead of HspAI; HpaII, BsiSI, and HapII instead of MspI), but their applicability should be verified experimentally. It should be noted that Figure 2, Figure 3, Figure 4 and Figure 5 only show results for the subspecies found in Russia.

The developed methods to identify species of honey bees by PCR-RFLP with mutagenic primers was tested with at least 9 samples of each subspecies. All the samples were correctly identified using this approach. However, it should be noted that, for more than 60% of the samples, amplification with highly specific AmCarp-f/AmCar-r primers was a necessary step prior PCR-RFLP.

We found that the *COX1* gene can be used as a marker for distinguishing honey bee subspecies. It showed no variability in *A. mellifera caucasica* and *A. mellifera carpatica* and very low variability in *A*. *mellifera carnica*. The high variability of *COX1* in *A. mellifera mellifera* is due to the existence of the two haplotypes of this subspecies (see above).

The data on the cytochrome b gene sequence in GenBank were limited and therefore insufficient for our purpose. We analyzed cytochrome b gene sequences in all four subspecies and found no SNPs typical for the individual subspecies [40].

The search for molecular genetic markers for the differentiation of subspecies in Russia is relevant. Despite the fact that, at the moment, the introgression of African populations of bees is not so acute in Russia because of the cold climate, the data obtained may be applicable to economically important populations of honey bees. The developed method can allow for the detection of the possible introgression of bees such as *A. m. intermissa* and *A. m. iberica*.

The developed method requires no sequencing and therefore eliminates the requirement for sequencing equipment and the transportation of samples to a sequencing facility, which might be time-consuming. Using our method, the identification of honey bee species can be performed in 4–6 h depending on the procedure used for DNA isolation. However, it should be noted that a general task for the future is the search for molecular genetic markers that are strictly associated with specific subspecies of honeybees and are very conservative markers inside the subspecies.

## 5. Conclusions

We analyzed the Folmer region of the *COX1* gene in honey bee subspecies cultivated at bee farms in Russia and identified subspecies-specific SNPs. We found that the *COX1* gene can be used as a marker for identification of honey bee subspecies used be beekeepers in Russia. We developed a method for the rapid identification of honey bee subspecies using PCR with mutagenic primers and restriction fragment length polymorphism (RFLP). The developed PCR-RFLP method for the identification of honey bee subspecies is convenient and inexpensive.

## Figures and Tables

**Table 1:** Morphometric parameters of studied honey bee breeds. M ± m—mean value and deviation from the mean value in the same units; C_v_—coefficient of feature variability.

Breed	Proboscis Length, mm		Third Tergit Width, mm		Cubital Index, %		Tarsal Index, %	
	M ± m	C_v_, %	M ± m	C_v_, %	M ± m	C_v_	M ± m	C_v_
*A. m. mellifera*	6.2 ± 0.02	1.8	5.0 ± 0.04	1.3	62.3 ± 1.5	6.2	55.6 ± 0.2	4.0
*A. m. carpatica*	6.7 ± 0.02	2.6	4.7 ± 0.01	2.2	43.1 ± 0.40	5.5	52.0 ± 0.6	2.5
*A. m. caucasica*	6.9 ± 0.01	1.2	4.7 ± 0.01	1.4	51.2 ± 0.20	3.2	55.0 ± 0.2	4.1
*A. m. carnica*	6.7 ± 0.02	2.2	4.9 ± 0.02	2.3	37.9 ± 0.3	5.0	54.0 ± 0.4	2.5

**Table 2:** Primers for amplification cytochrome oxidase subunit 1 and cytochrome b genes of mtDNA.

Primer Name	Primer Direction	Primer Sequence
LepF1	forward	ATTCAACCAATCATAAAGATATTGG
LepR1	reverse	TAAACTTCTGGATGTCCAAAAAATCA
AmCarp-f	forward	GAATATGAGCCGGAATAGTAGGA
AmCar-r	reverse	ATGTGTTGAAGTTACGGTCA
CYTB-f	forward	TATGTACTACCATGAGGACAAATATC
CYTB-r	reverse	ATTACACCTCCTAATTTATTAGGAAT

**Table 3:** Primers for honey bee subspecies identification.

Subspecies	Primers		5′–3′Sequence
*A. mellifera carnica*	AmCar-f	forward	ATTTCMTCAATTATAGGATCATTAAAYTTACC *
	AmCar-r	reverse	CAGCTAATACAGGTAATGA
*A. mellifera carpatica*	AmCarp-f	forward	AGATATTGGGATCTTGTA
	AmCarp-r	reverse	CTAGTAACAATTGTATTATAAATTTGATCAGCG *
*A. mellifera mellifera* H1	AmEu1-f	forward	GGATGAACAGTATATCCACC
	AmEu1-r	reverse	GTAACTATTAAGTTTAATGATCCTATAATAGC *
*A. mellifera mellifera* H2	AmEu2-f	forward	CTTTAATACTAGGATCACCTGATATAGCGAT *
	AmEu2-r	reverse	CTGATAATGGTGGATATA

H1: haplotype 1; H2: haplotype 2; * mutagenic primer.

**Table 4:** DNA fragments (bp) obtained by PCR-RLFP.

Primers	Restriction Endonuclease	Product Length, Bp				
		*A. mellifera mellifera* H1	*A. mellifera mellifera* H2	*A. mellifera carpatica*	*A. mellifera carnica*	*A. mellifera caucasica*
AmEu1-f, AmEu1-r	Alu I	107, 32	139	139	139	139
AmEu2-f, AmEu2-r.	Hinf I	150	122, 28	150	150	150
AmCarp-f, AmCarp-r	HspA I	141	141	111, 30	111, 30	141
AmCar-f, AmCar-r	Msp I	148	148	148	118, 30	148

H1: haplotype 1; H2: haplotype 2.

## Supplementary Materials

The following are available online at http://www.mdpi.com/2075-4450/9/1/10/s1, Nucleotide sequences of *Apis mellifera* subspecies cytochrome oxidase subunit 1 gene obtained during sequencing.
