# Supplementary material for: A Molecular Method for the Identification of Honey Bee Subspecies Used by Beekeepers in Russia
Source: Insects. 2018 Jan 27;9(1):10. doi: 10.3390/insects9010010 (PMC5872275; doi:10.3390/insects9010010)
Supplement: Supplementary file 1 [file insects-09-00010-s001.pdf]

insects
MDPI

Article

A Molecular Method for the Identification of Honey Bee Subspecies Used by Beekeepers in Russia

Supplementary Materials:

Nucleotide sequences of *Apis mellifera* subspecies cytochrome oxidase subunit 1 gene obtained during sequencing

>Seq1 [organism=Apis mellifera caucasica] Apis mellifera caucasica voucher VSU_1, cytochrome oxidase subunit 1 (COI) gene, partial cds; mitochondrial
ATTATTCGAATAGAATTAAGATCCCCAGGATCATGAATTAACAATGATCAAATTTATAATAC
AATTGTTACTAGTCATGCATTCCTAATAATTTTTTTTATAGTTATACCATTTTTAATTGGAGG
ATTTGGAAATTGGCTTATTCCTTTAATACTAGGATCACCTGATATAGCATTCCCCCGAATAA
ATAATATTAGATTTTGATTACTTCCTCCCTCATTATTTATACTTTTATTAAGAAATTTATTTTA
TCCAAGACCAGGAACTGGATGAACAGTATATCCACCATTATCAGCATATTTATATCATTCTT
CACCTTCAGTAGATTTTGCAATTTTTTCTCTTCATATATCAGGAATTTCCTCAATTATAGGAT
CATTAAACTTAATAGTTACAATTATAATAATAAAAAATTTTTCTATAAATTATGACCAAATT
>Seq2 [organism=Apis mellifera caucasica] Apis mellifera caucasica voucher VSU_2, cytochrome oxidase subunit 1 (COI) gene, partial cds; mitochondrial
ATTATTCGAATAGAATTAAGATCCCCAGGATCATGAATTAACAATGATCAAATTTATAATAC
AATTGTTACTAGTCATGCATTCCTAATAATTTTTTTTATAGTTATACCATTTTTAATTGGAGG
ATTTGGAAATTGGCTTATTCCTTTAATACTAGGATCACCTGATATAGCATTCCCCCGAATAA
ATAATATTAGATTTTGATTACTTCCTCCCTCATTATTTATACTTTTATTAAGAAATTTATTTTA
TCCAAGACCAGGAACTGGATGAACAGTATATCCACCATTATCAGCATATTTATATCATTCTT
CACCTTCAGTAGATTTTGCAATTTTTTCTCTTCATATATCAGGAATTTCCTCAATTATAGGAT
CATTAAACTTAATAGTTACAATTATAATAATAAAAAATTTTTCTATAAATTATGACCAAATT
>Seq3 [organism=Apis mellifera caucasica] Apis mellifera caucasica voucher VSU_3, cytochrome oxidase subunit 1 (COI) gene, partial cds; mitochondrial
ATTATTCGAATAGAATTAAGATCCCCAGGATCATGAATTAACAATGATCAAATTTATAATAC
AATTGTTACTAGTCATGCATTCCTAATAATTTTTTTTATAGTTATACCATTTTTAATTGGAGG
ATTTGGAAATTGGCTTATTCCTTTAATACTAGGATCACCTGATATAGCATTCCCCCGAATAA
ATAATATTAGATTTTGATTACTTCCTCCCTCATTATTTATACTTTTATTAAGAAATTTATTTTA
TCCAAGACCAGGAACTGGATGAACAGTATATCCACCATTATCAGCATATTTATATCATTCTT
CACCTTCAGTAGATTTTGCAATTTTTTCTCTTCATATATCAGGAATTTCCTCAATTATAGGAT
CATTAAACTTAATAGTTACAATTATAATAATAAAAAATTTTTCTATAAATTATGACCAAATT
>Seq4 [organism=Apis mellifera caucasica] Apis mellifera caucasica voucher VSU_4, cytochrome oxidase subunit 1 (COI) gene, partial cds; mitochondrial

ATTATTCGAATAGAATTAAGATCCCCAGGATCATGAATTAACAATGATCAAATTTATAATAC
AATTGTTACTAGTCATGCATTCCTAATAATTTTTTTTATAGTTATACCATTTTTAATTGGAGG
ATTTGGAAATTGGCTTATTCCTTTAATACTAGGATCACCTGATATAGCATTCCCCCGAATAA
ATAATATTAGATTTTGATTACTTCCTCCCTCATTATTTATACTTTTATTAAGAAATTTATTTTA
TCCAAGACCAGGAACTGGATGAACAGTATATCCACCATTATCAGCATATTTATATCATTCTT
CACCTTCAGTAGATTTTGCAATTTTTTCTCTTCATATATCAGGAATTTCCTCAATTATAGGAT
CATTAAACTTAATAGTTACAATTATAATAATAAAAAATTTTTCTATAAATTATGACCAAATT
>Seq5 [organism=Apis mellifera caucasica] Apis mellifera caucasica voucher VSU_5, cytochrome oxidase subunit 1 (COI) gene, partial cds; mitochondrial
ATTATTCGAATAGAATTAAGATCCCCAGGATCATGAATTAACAATGATCAAATTTATAATAC
AATTGTTACTAGTCATGCATTCCTAATAATTTTTTTTATAGTTATACCATTTTTAATTGGAGG
ATTTGGAAATTGGCTTATTCCTTTAATACTAGGATCACCTGATATAGCATTCCCCCGAATAA
ATAATATTAGATTTTGATTACTTCCTCCCTCATTATTTATACTTTTATTAAGAAATTTATTTTA
TCCAAGACCAGGAACTGGATGAACAGTATATCCACCATTATCAGCATATTTATATCATTCTT
CACCTTCAGTAGATTTTGCAATTTTTTCTCTTCATATATCAGGAATTTCCTCAATTATAGGAT
CATTAAACTTAATAGTTACAATTATAATAATAAAAAATTTTTCTATAAATTATGACCAAATT
>Seq6 [organism=Apis mellifera caucasica] Apis mellifera caucasica voucher VSU_6, cytochrome oxidase subunit 1 (COI) gene, partial cds; mitochondrial
ATTATTCGAATAGAATTAAGATCCCCAGGATCATGAATTAACAATGATCAAATTTATAATAC
AATTGTTACTAGTCATGCATTCCTAATAATTTTTTTTATAGTTATACCATTTTTAATTGGAGG
ATTTGGAAATTGGCTTATTCCTTTAATACTAGGATCACCTGATATAGCATTCCCCCGAATAA
ATAATATTAGATTTTGATTACTTCCTCCCTCATTATTTATACTTTTATTAAGAAATTTATTTTA
TCCAAGACCAGGAACTGGATGAACAGTATATCCACCATTATCAGCATATTTATATCATTCTT
CACCTTCAGTAGATTTTGCAATTTTTTCTCTTCATATATCAGGAATTTCCTCAATTATAGGAT
CATTAAACTTAATAGTTACAATTATAATAATAAAAAATTTTTCTATAAATTATGACCAAATT
>Seq7 [organism=Apis mellifera caucasica] Apis mellifera caucasica voucher VSU_7, cytochrome oxidase subunit 1 (COI) gene, partial cds; mitochondrial
ATTATTCGAATAGAATTAAGATCCCCAGGATCATGAATTAACAATGATCAAATTTATAATAC
AATTGTTACTAGTCATGCATTCCTAATAATTTTTTTTATAGTTATACCATTTTTAATTGGAGG
ATTTGGAAATTGGCTTATTCCTTTAATACTAGGATCACCTGATATAGCATTCCCCCGAATAA
ATAATATTAGATTTTGATTACTTCCTCCCTCATTATTTATACTTTTATTAAGAAATTTATTTTA
TCCAAGACCAGGAACTGGATGAACAGTATATCCACCATTATCAGCATATTTATATCATTCTT
CACCTTCAGTAGATTTTGCAATTTTTTCTCTTCATATATCAGGAATTTCCTCAATTATAGGAT
CATTAAACTTAATAGTTACAATTATAATAATAAAAAATTTTTCTATAAATTATGACCAAATT
>Seq8 [organism=Apis mellifera caucasica] Apis mellifera caucasica voucher VSU_8, cytochrome oxidase subunit 1 (COI) gene, partial cds; mitochondrial
ATTATTCGAATAGAATTAAGATCCCCAGGATCATGAATTAACAATGATCAAATTTATAATAC
AATTGTTACTAGTCATGCATTCCTAATAATTTTTTTTATAGTTATACCATTTTTAATTGGAGG
ATTTGGAAATTGGCTTATTCCTTTAATACTAGGATCACCTGATATAGCATTCCCCCGAATAA

ATAATATTAGATTTTGATTACTTCCTCCCTCATTATTTATACTTTTATTAAGAAATTTATTTTA
TCCAAGACCAGGAACTGGATGAACAGTATATCCACCATTATCAGCATATTTATATCATTCTT
CACCTTCAGTAGATTTTGCAATTTTTTCTCTTCATATATCAGGAATTTCCTCAATTATAGGAT
CATTAAACTTAATAGTTACAATTATAATAATAAAAAATTTTTCTATAAATTATGACCAAATT
>Seq9 [organism=Apis mellifera caucasica] Apis mellifera caucasica voucher VSU_9,
cytochrome oxidase subunit 1 (COI) gene, partial cds; mitochondrial
ATTATTCGAATAGAATTAAGATCCCCAGGATCATGAATTAACAATGATCAAATTTATAATAC
AATTGTTACTAGTCATGCATTCCTAATAATTTTTTTTATAGTTATACCATTTTTAATTGGAGG
ATTTGGAAATTGGCTTATTCCTTTAATACTAGGATCACCTGATATAGCATTCCCCCGAATAA
ATAATATTAGATTTTGATTACTTCCTCCCTCATTATTTATACTTTTATTAAGAAATTTATTTTA
TCCAAGACCAGGAACTGGATGAACAGTATATCCACCATTATCAGCATATTTATATCATTCTT
CACCTTCAGTAGATTTTGCAATTTTTTCTCTTCATATATCAGGAATTTCCTCAATTATAGGAT
CGATTAAACTTAATAGT
>Seq10 [organism=Apis mellifera caucasica] Apis mellifera caucasica voucher VSU_10,
cytochrome oxidase subunit 1 (COI) gene, partial cds; mitochondrial
ATTATTCGAATAGAATTAAGATCCCCAGGATCATGAATTAACAATGATCAAATTTATAATAC
AATTGTTACTAGTCATGCATTCCTAATAATTTTTTTTATAGTTATACCATTTTTAATTGGAGG
ATTTGGAAATTGGCTTATTCCTTTAATACTAGGATCACCTGATATAGCATTCCCCCGAATAA
ATAATATTAGATTTTGATTACTTCCTCCCTCATTATTTATACTTTTATTAAGAAATTTATTTTA
TCCAAGACCAGGAACTGGATGAACAGTATATCCACCATTATCAGCATATTTATATCATTCTT
CACCTTCAGTAGATTTTGCAATTTTTTCTCTTCATATATCAGGAATTTCCTCAATTATAGGAT
CATTAAACTTAATAGTTACAATTATAATAATAAAAAATTTTTCTATAAATTATGACCAAATT
>Seq11 [organism=Apis mellifera caucasica] Apis mellifera caucasica voucher VSU_11,
cytochrome oxidase subunit 1 (COI) gene, partial cds; mitochondrial
ATTATTCGAATAGAATTAAGATCCCCAGGATCATGAATTAACAATGATCAAATTTATAATAC
AATTGTTACTAGTCATGCATTCCTAATAATTTTTTTTATAGTTATACCATTTTTAATTGGAGG
ATTTGGAAATTGGCTTATTCCTTTAATACTAGGATCACCTGATATAGCATTCCCCCGAATAA
ATAATATTAGATTTTGATTACTTCCTCCCTCATTATTTATACTTTTATTAAGAAATTTATTTTA
TCCAAGACCAGGAACTGGATGAACAGTATATCCACCATTATCAGCATATTTATATCATTCTT
CACCTTCAGTAGATTTTGCAATTTTTTCTCTTCATATATCAGGAATTTCCTCAATTATAGGAT
CATTAAACTTAATAGTTACAATTATAATAATAAAAAATTTTTCTATAAATTATGACCAAATT
>Seq12 [organism=Apis mellifera carnica] Apis mellifera carnica voucher VSU_12, cytochrome
oxidase subunit 1 (COI) gene, partial cds; mitochondrial
ATTATTCGAATAGAATTAAGATCCCCAGGATCATGAATTAGCAATGATCAAATTTATAATAC
AATTGTTACTAGTCATGCATTCCTAATAATTTTTTTTATAGTTATACCATTTTTAATTGGAGG
ATTTGGAAATTGGCTTATTCCTTTAATACTAGGATCACCTGATATAGCATTCCCCCGAATAA
ATAATATTAGATTTTGATTACTTCCTCCCTCATTATTTATACTTTTATTAAGAAATTTATTTTA
TCCAAGACCAGGAACTGGATGAACAGTATATCCACCATTATCAGCATATTTATATCATTCTT

CACCTTCAGTAGATTTTGCAATTTTTTCTCTTCATATATCAGGAATTTCCTCAATTATAGGAT
CATTAAACTTAATGGTTACAATTATAATAATAAAAAATTTTTCTATAAATTATGACCAAATT
>Seq13 [organism=Apis mellifera carnica] Apis mellifera carnica voucher VSU_13, cytochrome oxidase subunit 1 (COI) gene, partial cds; mitochondrial
ATTATTCGAATAGAATTAAGATCCCCAGGATCATGAATTAGCAATGATCAAATTTATAATAC
AATTGTTACTAGTCATGCATTCCTAATAATTTTTTTTATAGTTATACCATTTTTAATTGGAGG
ATTTGGAAATTGGCTTATTCCTTTAATACTAGGATCACCTGATATAGCATTCCCCCGAATAA
ATAATATTAGATTTTGATTACTTCCTCCCTCATTATTTATACTTTTATTAAGAAATTTATTTTA
TCCAAGACCAGGAACTGGATGAACAGTATATCCACCATTATCAGCATATTTATATCATTCTT
CACCTTCAGTAGATTTTGCAATTTTTTCTCTTCATATATCAGGAATTTCCTCAATTATAGGAT
CATTAAACTTAATAGTTACAATTATAATAATAAAAAATTTTTCTATAAATTATGACCAAATT
>Seq14 [organism=Apis mellifera carnica] Apis mellifera carnica voucher VSU_14, cytochrome oxidase subunit 1 (COI) gene, partial cds; mitochondrial
ATTATTCGAATAGAATTAAGATCCCCAGGATCATGAATTAGCAATGATCAAATTTATAATAC
AATTGTTACTAGTCATGCATTCCTAATAATTTTTTTTATAGTTATACCATTTTTAATTGGAGG
ATTTGGAAATTGGCTTATTCCTTTAATACTAGGATCACCTGATATAGCATTCCCCCGAATAA
ATAATATTAGATTTTGATTACTTCCTCCCTCATTATTTATACTTTTATTAAGAAATTTATTTTA
TCCAAGACCAGGAACTGGATGAACAGTATATCCACCATTATCAGCATATTTATATCATTCTT
CACCTTCAGTAGATTTTGCAATTTTTTCTCTTCATATATCAGGAATTTCCTCAATTATAGGAT
CATTAAACTTAATGGTTACAATTATAATAATAAAAAATTTTTCTATAAATTATGACCAAATT
>Seq15 [organism=Apis mellifera carnica] Apis mellifera carnica voucher VSU_15, cytochrome oxidase subunit 1 (COI) gene, partial cds; mitochondrial
ATTATTCGAATAGAATTAAGATCCCCAGGATCATGAATTAGCAATGATCAAATTTATAATAC
AATTGTTACTAGTCATGCATTCCTAATAATTTTTTTTATAGTTATACCATTTTTAATTGGAGG
ATTTGGAAATTGGCTTATTCCTTTAATACTAGGATCACCTGATATAGCATTCCCCCGAATAA
ATAATATTAGATTTTGATTACTTCCTCCCTCATTATTTATACTTTTATTAAGAAATTTATTTTA
TCCAAGACCAGGAACTGGATGAACAGTATATCCACCATTATCAGCATATTTATATCATTCTT
CACCTTCAGTAGATTTTGCAATTTTTTCTCTTCATATATCAGGAATTTCCTCAATTATAGGAT
CATTAAACTTAATAGTTACAATTATAATAATAAAAAATTTTTCTATAAATTATGACCAAATT
>Seq16 [organism=Apis mellifera carnica] Apis mellifera carnica voucher VSU_16, cytochrome oxidase subunit 1 (COI) gene, partial cds; mitochondrial
ATTATTCGAATAGAATTAAGATCCCCAGGATCATGAATTAGCAATGATCAAATTTATAATAC
AATTGTTACTAGTCATGCATTCCTAATAATTTTTTTTATAGTTATACCATTTTTAATTGGAGG
ATTTGGAAATTGGCTTATTCCTTTAATACTAGGATCACCTGATATAGCATTCCCCCGAATAA
ATAATATTAGATTTTGATTACTTCCTCCCTCATTATTTATACTTTTATTAAGAAATTTATTTTA
TCCAAGACCAGGAACTGGATGAACAGTATATCCACCATTATCAGCATATTTATATCATTCTT
CACCTTCAGTAGATTTTGCAATTTTTTCTCTTCATATATCAGGAATTTCCTCAATTATAGGAT
CATTAAACTTAATGGTTACAATTATAATAATAAAAAATTTTTCTATAAATTATGACCAAATT

>Seq17 [organism=Apis mellifera carnica] Apis mellifera carnica voucher VSU_17, cytochrome oxidase subunit 1 (COI) gene, partial cds; mitochondrial
ATTATTCGAATAGAATTAAGATCCCCAGGATCATGAATTAGCAATGATCAAATTTATAATAC
AATTGTTACTAGTCATGCATTCCTAATAATTTTTTTTATAGTTATACCATTTTTAATTGGAGG
ATTTGGAAATTGGCTTATTCCTTTAATACTAGGATCACCTGATATAGCATTCCCCCGAATAA
ATAATATTAGATTTTGATTACTTCCTCCCTCATTATTTATACTTTTATTAAGAAATTTATTTTA
TCCAAGACCAGGAACTGGATGAACAGTATATCCACCATTATCAGCATATTTATATCATTCTT
CACCTTCAGTAGATTTTGCAATTTTTTCTCTTCATATATCAGGAATTTCCTCAATTATAGGAT
CATTAAACTTAATGGTTACAATTATAATAATAAAAAATTTTTCTATAAATTATGACCAAATT
>Seq18 [organism=Apis mellifera carnica] Apis mellifera carnica voucher VSU_18, cytochrome oxidase subunit 1 (COI) gene, partial cds; mitochondrial
ATTATTCGAATAGAATTAAGATCCCCAGGATCATGAATTAGCAATGATCAAATTTATAATAC
AATTGTTACTAGTCATGCATTCCTAATAATTTTTTTTATAGTTATACCAATTTTAATTGGAGG
ATTTGGAAATTGGCTTATTCCTTTAATACTAGGATCACCTGATATAGCATTCCCCCGAATAA
ATAATATTAGATTTTGATTACTTCCTCCCTCATTATTTATACTTTTATTAAGAAATTTATTTTA
TCCAAGACCAGGAACTGGATGAACAGTATATCCACCATTATCAGCATATTTATATCATTCTT
CACCTTCAGTAGATTTTGCAATTTTTTCTCTTCATATATCAGGAATTTCCTCAATTATAGGAT
CATTAAACTTAATGGTTACAATTATAATAATAAAAAATTTTTCTATAAATTATGACCAAATT
>Seq19 [organism=Apis mellifera carnica] Apis mellifera carnica voucher VSU_19, cytochrome oxidase subunit 1 (COI) gene, partial cds; mitochondrial
ATTATTCGAATAGAATTAAGATCCCCAGGATCATGAATTAGCAATGATCAAATTTATAATAC
AATTGTTACTAGTCATGCATTCCTAATAATTTTTTTTATAGTTATACCATTTTTAATTGGAGG
ATTTGGAAATTGGCTTATTCCTTTAATACTAGGATCACCTGATATAGCATTCCCCCGAATAA
ATAATATTAGATTTTGATTACTTCCTCCCTCATTATTTATACTTTTATTAAGAAATTTATTTTA
TCCAAGACCAGGAACTGGATGAACAGTATATCCACCATTATCAGCATATTTATATCATTCTT
CACCTTCAGTAGATTTTGCAATTTTTTCTCTTCATATATCAGGAATTTCCTCAATTATAGGAT
CATTAAACTTAATGGTTACAATTATAATAATAAAAAATTTTTCTATAAATTATGACCAAATT
>Seq20 [organism=Apis mellifera carnica] Apis mellifera carnica voucher VSU_20, cytochrome oxidase subunit 1 (COI) gene, partial cds; mitochondrial
ATTATTCGAATAGAATTAAGATCCCCAGGATCATGAATTAGCAATGATCAAATTTATAATAC
AATTGTTACTAGTCATGCATTCCTAATAATTTTTTTTATAGTTATACCATTTTTAATTGGAGG
ATTTGGAAATTGGCTTATTCCTTTAATACTAGGATCACCTGATATAGCATTCCCCCGAATAA
ATAATATTAGATTTTGATTACTTCCTCCCTCATTATTTATACTTTTATTAAGAAATTTATTTTA
TCCAAGACCAGGAACTGGATGAACAGTATATCCACCATTATCAGCATATTTATATCATTCTT
CACCTTCAGTAGATTTTGCAATTTTTTCTCTTCATATATCAGGAATTTCCTCAATTATAGGAT
CATTAAACTTAATGGTTACAATTATAATAATAAAAAATTTTTCTATAAATTATGACCAAATT
>Seq21 [organism=Apis mellifera carnica] Apis mellifera carnica voucher VSU_21, cytochrome oxidase subunit 1 (COI) gene, partial cds; mitochondrial

ATTATTCGAATAGAATTAAGATCCCCAGGATCATGAATTAGCAATGATCAAATTTATAATAC
AATTGTTACTAGTCATGCATTCCTAATAATTTTTTTTATAGTTATACCATTTTTAATTGGAGG
ATTTGGAAATTGGCTTATTCCTTTAATACTAGGATCACCTGATATAGCATTCCCCCGAATAA
ATAATATTAGATTTTGATTACTTCCTCCCTCATTATTTATACTTTTATTAAGAAATTTATTTTA
TCCAAGACCAGGAACTGGATGAACAGTATATCCACCATTATCAGCATATTTATATCATTCTT
CACCTTCAGTAGATTTTGCAATTTTTTCTCTTCATATATCAGGAATTTCCTCAATTATAGGAT
CATTAAACTTAATGGTTACAATTATAATAATAAAAAATTTTTCTATAAATTATGACCAAATT
>Seq22 [organism=Apis mellifera carnica] Apis mellifera carnica voucher VSU_22, cytochrome oxidase subunit 1 (COI) gene, partial cds; mitochondrial
ATTATTCGAATAGAATTAAGATCCCCAGGATCATGAATTAGCAATGATCAAATTTATAATAC
AATTGTTACTAGTCATGCATTCCTAATAATTTTTTTTATAGTTATACCAAAATTAATTGGAGG
ATTTGGAAATTGGCTTATTCCTTTAATACTAGGATCACCTGATATAGCATTCCCCCGAATAA
ATAATATTAGATTTTGATTACTTCCTCCCTCATTATTTATACTTTTATTAAGAAATTTATTTTA
TCCAAGACCAGGAACTGGATGAACAGTATATCCACCATTATCAGCATATTTATATCATTCTT
CACCTTCAGTAGATTTTGCAATTTTTTCTCTTCATATATCAGGAATTTCCTCAATTATAGGAT
CATTAAACTTAATGGTTACAATTATAATAATAAAAAATTTTTCTATAAATTATGACCAAATT
>Seq23 [organism=Apis mellifera carnica] Apis mellifera carnica voucher VSU_23, cytochrome oxidase subunit 1 (COI) gene, partial cds; mitochondrial
ATTATTCGAATAGAATTAAGATCCCCAGGATCATGAATTAGCAATGATCAAATTTATAATAC
AATTGTTACTAGTCATGCATTCCTAATAATTTTTTTTATAGTTATACCATTTTTAATTGGAGG
ATTTGGAAATTGGCTTATTCCTTTAATACTAGGATCACCTGATATAGCATTCCCCCGAATAA
ATAATATTAGATTTTGATTACTTCCTCCCTCATTATTTATACTTTTATTAAGAAATTTATTTTA
TCCAAGACCAGGAACTGGATGAACAGTATATCCACCATTATCAGCATATTTATATCATTCTT
CACCTTCAGTAGATTTTGCAATTTTTTCTCTTCATATATCAGGAATTTCCTCAATTATAGGAT
CATTAAACTTAATGGTTACTTTTATAATAATAAAAAATTTTTCTATAAATTATGACCAAATT
>Seq24 [organism=Apis mellifera carnica] Apis mellifera carnica voucher VSU_24, cytochrome oxidase subunit 1 (COI) gene, partial cds; mitochondrial
ATTATTCGAATAGAATTAAGATCCCCAGGATCATGAATTAGCAATGATCAAATTTATAATACAAT
TGTTACTAGTCATGCATTCCTAATAATTTTTTTTATAGTTATACCATTTTTAATTGGAGGATTTGG
AAATTGGCTTATTCCTTTAATACTAGGATCACCTGATATAGCATTCCCCCGAATAAATAATATTA
GATTTTGATTACTTCCTCCCTCATTATTTATACTTTTATTAAGAAATTTATTTTATCCAAGACCAG
GAACTGGATGAACAGTATATCCACCATTATCAGCATATTTATATCATTCTTCACCTTCAGTAGAT
TTTGCAATTTTTTCTCTTCATATATCAGGAATTTCCTCAATTATAGGATCATTAAACTTAATGGTT
ACAATTATAATAATAAAAAATTTTTCTATAAATTATGACCAAATT
>Seq25 [organism=Apis mellifera carnica] Apis mellifera carnica voucher VSU_25, cytochrome oxidase subunit 1 (COI) gene, partial cds; mitochondrial
ATTATTCGAATAGAATTAAGATCCCCAGGATCATGAATTAGCAATGATCAAATTTATAATAC
AATTGTTACTAGTCATGCATTCCTAATAATTTTTTTTATAGTTATACCATTTTTAATTGGAGG
ATTTGGAAATTGGCTTATTCCTTTAATACTAGGATCACCTGATATAGCATTCCCCCGAATAA
ATAATATTAGATTTTGATTACTTCCTCCCTCATTATTTATACTTTTATTAAGAAATTTATTTTA

TCCAAGACCAGGAACTGGATGAACAGTATATCCACCATTATCAGCATATTTATATCATTCTT
CACCTTCAGTAGATTTTGCAATTTTTTCTCTTCATATATCAGGAATTTCCTCAATTATAGGAT
CATTAAACTTAATGGTTACAATTATAATAATAAAAAATTTTTCTATAAATTATGACCAAATT
>Seq26 [organism=Apis mellifera carnica] Apis mellifera carnica voucher VSU_26, cytochrome oxidase subunit 1 (COI) gene, partial cds; mitochondrial
ATTATTCGAATAGAATTAAGATCCCCAGGATCATGAATTAGCAATGATCAAATTTATAATAC
AATTGTTACTAGTCATGCATTCCTAATAATTTTTTTTATAGTTATACCATTTTTAATTGGAGG
ATTTGGAAATTGGCTTATTCCTTTAATACTAGGATCACCTGATATAGCATTCCCCCGAATAA
ATAATATTAGATTTTGATTACTTCCTCCCTCATTATTTATACTTTTATTAAGAAATTTATTTTA
TCCAAGACCAGGAACTGGATGAACAGTATATCCACCATTATCAGCATATTTATATCATTCTT
CACCTTCAGTAGATTTTGCAATTTTTTCTCTTCATATATCAGGAATTTCCTCAATTATAGGAT
CATTAAACTTAATGGTTACAATTATAATAATAAAAAATTTTTCTATAAATTATGACCAAATT
>Seq27 [organism=Apis mellifera carnica] Apis mellifera carnica voucher VSU_27, cytochrome oxidase subunit 1 (COI) gene, partial cds; mitochondrial
ATTATTCGAATAGAATTAAGATCCCCAGGATCATGAATTAGCAATGATCAAATTTATAATAC
AATTGTTACTAGTCATGCATTCCTAATAATTTTTTTTATAGTTATACCATTTTTAATTGGAGG
ATTTGGAAATTGGCTTATTCCTTTAATACTAGGATCACCTGATATAGCATTCCCCCGAATAA
ATAATATTAGATTTTGATTACTTCCTCCCTCATTATTTATACTTTTATTAAGAAATTTATTTTA
TCCAAGACCAGGAACTGGATGAACAGTATATCCACCATTATCAGCATATTTATATCATTCTT
CACCTTCAGTAGATTTTGCAATTTTTTCTCTTCATATATCAGGAATTTCCTCAATTATAGGAT
CATTAAACTTAATGGTTACAATTATAATAATAAAAAATTTTTCTATAAATTATGACCAAATT
>Seq28 [organism=Apis mellifera carpatica] Apis mellifera carpatica voucher VSU_28, cytochrome oxidase subunit 1 (COI) gene, partial cds; mitochondrial
ATTATTCGAATAGAATTAAGATCCCCAGGATCATGAATTAGCAATGATCAAATTTATAATAC
AATTGTTACTAGTCATGCATTCCTAATAATTTTTTTTATAGTTATACCATTTTTAATTGGAGG
ATTTGGAAATTGGCTTATTCCTTTAATACTAGGATCACCTGATATAGCATTCCCCCGAATAA
ATAATATTAGATTTTGATTACTTCCTCCCTCATTATTTATACTTTTATTAAGAAATTTATTTTA
TCCAAGACCAGGAACTGGATGAACAGTATATCCACCATTATCAGCATATTTATATCATTCTT
CACCTTCAGTAGATTTTGCAATTTTTTCTCTTCATATATCAGGAATTTCCTCAATTATAGGAT
CATTAAACTTAATAGTTACAATTATAATAATAAAAAATTTTTCTATAAATTATGACCAAATT
>Seq29 [organism=Apis mellifera carpatica] Apis mellifera carpatica voucher VSU_29, cytochrome oxidase subunit 1 (COI) gene, partial cds; mitochondrial
ATTATTCGAATAGAATTAAGATCCCCAGGATCATGAATTAGCAATGATCAAATTTATAATAC
AATTGTTACTAGTCATGCATTCCTAATAATTTTTTTTATAGTTATACCATTTTTAATTGGAGG
ATTTGGAAATTGGCTTATTCCTTTAATACTAGGATCACCTGATATAGCATTCCCCCGAATAA
ATAATATTAGATTTTGATTACTTCCTCCCTCATTATTTATACTTTTATTAAGAAATTTATTTTA
TCCAAGACCAGGAACTGGATGAACAGTATATCCACCATTATCAGCATATTTATATCATTCTT
CACCTTCAGTAGATTTTGCAATTTTTTCTCTTCATATATCAGGAATTTCCTCAATTATAGGAT
CATTAAACTTAATAGTTACAATTATAATAATAAAAAATTTTTCTATAAATTATGACCAAATT

>Seq30 [organism=Apis mellifera carpatica] Apis mellifera carpatica voucher VSU_30, cytochrome oxidase subunit 1 (COI) gene, partial cds; mitochondrial
ATTATTCGAATAGAATTAAGATCCCCAGGATCATGAATTAGCAATGATCAAATTTATAATAC
AATTGTTACTAGTCATGCATTCCTAATAATTTTTTTTATAGTTATACCATTTTTAATTGGAGG
ATTTGGAAATTGGCTTATTCCTTTAATACTAGGATCACCTGATATAGCATTCCCCCGAATAA
ATAATATTAGATTTTGATTACTTCCTCCCTCATTATTTATACTTTTATTAAGAAATTTATTTTA
TCCAAGACCAGGAACTGGATGAACAGTATATCCACCATTATCAGCATATTTATATCATTCTT
CACCTTCAGTAGATTTTGCAATTTTTTCTCTTCATATATCAGGAATTTCCTCAATTATAGGAT
CATTAAACTTAATAGTTACAATTATAATAATAAAAAATTTTTCTATAAATTATGACCAAATT
>Seq31 [organism=Apis mellifera carpatica] Apis mellifera carpatica voucher VSU_31, cytochrome oxidase subunit 1 (COI) gene, partial cds; mitochondrial
ATTATTCGAATAGAATTAAGATCCCCAGGATCATGAATTAGCAATGATCAAATTTATAATAC
AATTGTTACTAGTCATGCATTCCTAATAATTTTTTTTATAGTTATACCATTTTTAATTGGAGG
ATTTGGAAATTGGCTTATTCCTTTAATACTAGGATCACCTGATATAGCATTCCCCCGAATAA
ATAATATTAGATTTTGATTACTTCCTCCCTCATTATTTATACTTTTATTAAGAAATTTATTTTA
TCCAAGACCAGGAACTGGATGAACAGTATATCCACCATTATCAGCATATTTATATCATTCTT
CACCTTCAGTAGATTTTGCAATTTTTTCTCTTCATATATCAGGAATTTCCTCAATTATAGGAT
CATTAAACTTAATAGTTACAATTATAATAATAAAAAATTTTTCTATAAATTATGACCAAATT
>Seq32 [organism=Apis mellifera carpatica] Apis mellifera carpatica voucher VSU_32, cytochrome oxidase subunit 1 (COI) gene, partial cds; mitochondrial
ATTATTCGAATAGAATTAAGATCCCCAGGATCATGAATTAGCAATGATCAAATTTATAATAC
AATTGTTACTAGTCATGCATTCCTAATAATTTTTTTTATAGTTATACCATTTTTAATTGGAGG
ATTTGGAAATTGGCTTATTCCTTTAATACTAGGATCACCTGATATAGCATTCCCCCGAATAA
ATAATATTAGATTTTGATTACTTCCTCCCTCATTATTTATACTTTTATTAAGAAATTTATTTTA
TCCAAGACCAGGAACTGGATGAACAGTATATCCACCATTATCAGCATATTTATATCATTCTT
CACCTTCAGTAGATTTTGCAATTTTTTCTCTTCATATATCAGGAATTTCCTCAATTATAGGAT
CATTAAACTTAATAGTTACAATTATAATAATAAAAAATTTTTCTATAAATTATGACCAAATT
>Seq33 [organism=Apis mellifera carpatica] Apis mellifera carpatica voucher VSU_33, cytochrome oxidase subunit 1 (COI) gene, partial cds; mitochondrial
ATTATTCGAATAGAATTAAGATCCCCAGGATCATGAATTAGCAATGATCAAATTTATAATAC
AATTGTTACTAGTCATGCATTCCTAATAATTTTTTTTATAGTTATACCATTTTTAATTGGAGG
ATTTGGAAATTGGCTTATTCCTTTAATACTAGGATCACCTGATATAGCATTCCCCCGAATAA
ATAATATTAGATTTTGATTACTTCCTCCCTCATTATTTATACTTTTATTAAGAAATTTATTTTA
TCCAAGACCAGGAACTGGATGAACAGTATATCCACCATTATCAGCATATTTATATCATTCTT
CACCTTCAGTAGATTTTGCAATTTTTTCTCTTCATATATCAGGAATTTCCTCAATTATAGGAT
CATTAAACTTAATAGTTACAATTATAATAATAAAAAATTTTTCTATAAATTATGACCAAATT
>Seq34 [organism=Apis mellifera carpatica] Apis mellifera carpatica voucher VSU_34, cytochrome oxidase subunit 1 (COI) gene, partial cds; mitochondrial

ATTATTCGAATAGAATTAAGATCCCCAGGATCATGAATTAGCAATGATCAAATTTATAATAC
AATTGTTACTAGTCATGCATTCCTAATAATTTTTTTTATAGTTATACCATTTTTAATTGGAGG
ATTTGGAAATTGGCTTATTCCTTTAATACTAGGATCACCTGATATAGCATTCCCCCGAATAA
ATAATATTAGATTTTGATTACTTCCTCCCTCATTATTTATACTTTTATTAAGAAATTTATTTTA
TCCAAGACCAGGAACTGGATGAACAGTATATCCACCATTATCAGCATATTTATATCATTCTT
CACCTTCAGTAGATTTTGCAATTTTTTCTCTTCATATATCAGGAATTTCCTCAATTATAGGAT
CATTAAACTTAATAGTTACAATTATAATAATAAAAAATTTTTCTATAAATTATGACCAAATT
>Seq35 [organism=Apis mellifera carpatica] Apis mellifera carpatica voucher VSU_35, cytochrome oxidase subunit 1 (COI) gene, partial cds; mitochondrial
ATTATTCGAATAGAATTAAGATCCCCAGGATCATGAATTAGCAATGATCAAATTTATAATAC
AATTGTTACTAGTCATGCATTCCTAATAATTTTTTTTATAGTTATACCATTTTTAATTGGAGG
ATTTGGAAATTGGCTTATTCCTTTAATACTAGGATCACCTGATATAGCATTCCCCCGAATAA
ATAATATTAGATTTTGATTACTTCCTCCCTCATTATTTATACTTTTATTAAGAAATTTATTTTA
TCCAAGACCAGGAACTGGATGAACAGTATATCCACCATTATCAGCATATTTATATCATTCTT
CACCTTCAGTAGATTTTGCAATTTTTTCTCTTCATATATCAGGAATTTCCTCAATTATAGGAT
CATTAAACTTAATAGTTACAATTATAATAATAAAAAATTTTTCTATAAATTATGACCAAATT
>Seq36 [organism=Apis mellifera carpatica] Apis mellifera carpatica voucher VSU_36, cytochrome oxidase subunit 1 (COI) gene, partial cds; mitochondrial
ATTATTCGAATAGAATTAAGATCCCCAGGATCATGAATTAGCAATGATCAAATTTATAATAC
AATTGTTACTAGTCATGCATTCCTAATAATTTTTTTTATAGTTATACCATTTTTAATTGGAGG
ATTTGGAAATTGGCTTATTCCTTTAATACTAGGATCACCTGATATAGCATTCCCCCGAATAA
ATAATATTAGATTTTGATTACTTCCTCCCTCATTATTTATACTTTTATTAAGAAATTTATTTTA
TCCAAGACCAGGAACTGGATGAACAGTATATCCACCATTATCAGCATATTTATATCATTCTT
CACCTTCAGTAGATTTTGCAATTTTTTCTCTTCATATATCAGGAATTTCCTCAATTATAGGAT
CATTAAACTTAATAGTTACAATTATAATAATAAAAAATTTTTCTATAAATTATGACCAAATT
>Seq37 [organism=Apis mellifera carpatica] Apis mellifera carpatica voucher VSU_37, cytochrome oxidase subunit 1 (COI) gene, partial cds; mitochondrial
TTATTATTAAAAATAGAATTAAGATCCCCAGGATCATGAATTAGCAATGATCAAATTTATAAT
ACAATTGTTACTAGTCATGCATTCCTAATAATTTTTTTTATAGTTATACCATTTTTAATTGGA
GGATTTGGAAATTGGCTTATTCCTTTAATACTAGGATCACCTGATATAGCATTCCCCCGAAT
AAATAATATTAGATTTTGATTACTTCCTCCCTCATTATTTATACTTTTATTAAGAAATTTATTT
TATCCAAGACCAGGAACTGGATGAACAGTATATCCACCATTATCAGCATATTTATATCATTC
TTCACCTTCAGTAGATTTTGCAATTTTTTCTCTTCATATATCAGGAATTTCCTCAATTATAGG
ATCATTAAACTTAATAGTTACAATTATAATAATAAAAAATTTTTCTATAAATTATGACCAAATT
>Seq38 [organism=Apis mellifera carpatica] Apis mellifera carpatica voucher VSU_38, cytochrome oxidase subunit 1 (COI) gene, partial cds; mitochondrial
ATTATTCGAATAGAATTAAGATCCCCAGGATCATGAATTAGCAATGATCAAATTTATAATAC
AATTGTTACTAGTCATGCATTCCTAATAATTTTTTTTATAGTTATACCATTTTTAATTGGAGG
ATTTGGAAATTGGCTTATTCCTTTAATACTAGGATCACCTGATATAGCATTCCCCCGAATAA

ATAATATTAGATTTTGATTACTTCCTCCCTCATTATTTATACTTTTATTAAGAAATTTATTTTA
TCCAAGACCAGGAACTGGATGAACAGTATATCCACCATTATCAGCATATTTATATCATTCTT
CACCTTCAGTAGATTTTGCAATTTTTTCTCTTCATATATCAGGAATTTCCTCAATTATAGGAT
CATTAAACTTAATAGTTACAATTATAATAATAAAAAATTTTTCTATAAATTATGACCAAATT
>Seq39 [organism=Apis mellifera mellifera] Apis mellifera mellifera voucher VSU_39,
cytochrome oxidase subunit 1 (COI) gene, partial cds; mitochondrial
ATTATTTGAATAGAATTAAGATCTCCAGGATCATGAATTAATAACGATCAAATTTATAATACA
ATTGTTACTAGCCACGCATTTCTAATAATCTTTTTTATAGTTATACCATTTCTAATTGGAGGA
TTTGGAAATTGGCTTATTCCTTTAATACTAGGATCACCTGATATAGCATTTCCCCGAATAAA
TAATGTTAGATTTTGATTACTTCCTCCCTCATTATTAATACTTTTATTAAGAAATTTATTTTAC
CCAAGACCAGGAACTGGATGAACAGTATATCCACCATTATCAGCATATTTATATCACTCTT
CACCTTCAGTAGATTTTGCAATTTTTTCTCTTCATATATCAGGAATTTCCTCAATTATAGGAT
CATTAAATTTAATAGTTACAATTATAATAATAAAAAATTTTTCTATAAATTATGACCAAATT
>Seq40 [organism=Apis mellifera mellifera] Apis mellifera mellifera voucher VSU_40,
cytochrome oxidase subunit 1 (COI) gene, partial cds; mitochondrial
ATTATTCGAATAGAATTAAGATCCCCAGGATCATGAATTAACAATGATCAAATTTATAATAC
AATTGTTACTAGTCATGCATTCCTAATAATTTTTTTTATAGTTATACCATTTTTAATTGGAGG
ATTTGGAAATTGGCTTATTCCTTTAATACTAGGATCACCTGATATAGCATTCCCCCGAATAA
ATAATATTAGATTTTGATTACTTCCTCCCTCATTATTTATACTTTTATTAAGAAATTTATTTTA
TCCAAGACCAGGAACTGGATGAACAGTATATCCACCATTATCAGCATATTTATATCATTCTT
CACCTTCAGTAGATTTTGCAATTTTTTCTCTTCATATATCAGGAATTTCATCAATTATAGGAT
CATTAAACTTAATAGTTACAATTATAATAATAAAAAATTTTTCTATAAATTATGACCAAATT
>Seq41 [organism=Apis mellifera mellifera] Apis mellifera mellifera voucher VSU_41,
cytochrome oxidase subunit 1 (COI) gene, partial cds; mitochondrial
ATTATTCGAATAGAATTAAGATCTCCAGGATCATGAATTAATAACGATCAAATTTATAATAC
AATTGTTACTAGCCACGCATTTCTAATAATCTTTTTTATAGTTATACCATTTCTAATTGGAGG
ATTTGGAAATTGGCTTATTCCTTTAATACTAGGATCACCTGATATAGCATTTCCCCGAATAA
ATAATGTTAGATTTTGATTACTTCCTCCCTCATTATTAATACTTTTATTAAGAAATTTATTTTA
CCCAAGACCAGGAACTGGATGAACAGTATATCCACCATTATCAGCATATTTATATCACTCT
TCACCTTCAGTAGATTTTGCAATTTTTTCTCTTCATATATCAGGAATTTCCTCAATTATAGGA
TCATTAAATTTAATAGTTACAATTATAATAATAAAAAATTTTTCTATAAATTATGACCAAATT
>Seq42 [organism=Apis mellifera mellifera] Apis mellifera mellifera voucher VSU_42,
cytochrome oxidase subunit 1 (COI) gene, partial cds; mitochondrial
ATTATTCGAATAGAATTAAGATCCCCAGGATCATGAATTAACAATGATCAAATTTATAATAC
AATTGTTACTAGTCATGCATTCCTAATAATTTTTTTTATAGTTATACCATTTTTAATTGGAGG
ATTTGGAAATTGGCTTATTCCTTTAATACTAGGATCACCTGATATAGCATTCCCCCGAATAA
ATAATATTAGATTTTGATTACTTCCTCCCTCATTATTTATACTTTTATTAAGAAATTTATTTTA
TCCAAGACCAGGAACTGGATGAACAGTATATCCACCATTATCAGCATATTTATATCATTCTT

CACCTTCAGTAGATTTTGCAATTTTTTCTCTTCATATATCAGGAATTTCATCAATTATAGGAT
CATTAAACTTAATAGTTACAATTATAATAATAAAAAATTTTTCTATAAATTATGACCAAATT
>Seq43 [organism=Apis mellifera mellifera] Apis mellifera mellifera voucher VSU_43, cytochrome oxidase subunit 1 (COI) gene, partial cds; mitochondrial
ATTATTCGAATAGAATTAAGATCCCCAGGATCATGAATTAACAATGATCAAATTTATAATAC
AATTGTTACTAGTCATGCATTCCTAATAATTTTTTTTATAGTTATACCATTTTTAATTGGAGG
ATTTGGAAATTGGCTTATTCCTTTAATACTAGGATCACCTGATATAGCATTCCCCCGAATAA
ATAATATTAGATTTTGATTACTTCCTCCCTCATTATTTATACTTTTATTAAGAAATTTATTTTA
TCCAAGACCAGGAACTGGATGAACAGTATATCCACCATTATCAGCATATTTATATCATTCTT
CACCTTCAGTAGATTTTGCAATTTTTTCTCTTCATATATCAGGAATTTCATCAATTATAGGAT
CATTAAACTTAATAGTTACAATTATAATAATAAAAAATTTTTCTATAAATTATGACCAAATT
>Seq44 [organism=Apis mellifera mellifera] Apis mellifera mellifera voucher VSU_44, cytochrome oxidase subunit 1 (COI) gene, partial cds; mitochondrial
ATTATTCGAATAGAATTAAGATCTCCAGGATCATGAATTAATAACGATCAAATTTATAATAC
AATTGTTACTAGCCACGCATTTCTAATAATCTTTTTTATAGTTATACCATTTCTAATTGGAGG
ATTTGGAAATTGGCTTATTCCTTTAATACTAGGATCACCTGATATAGCATTTCCCCGAATAA
ATAATGTTAGATTTTGATTACTTCCTCCCTCATTATTAATACTTTTATTAAGAAATTTATTTTA
CCCAAGACCAGGAACTGGATGAACAGTATATCCACCATTATCAGCATATTTATATCACTCT
TCACCTTCAGTAGATTTTGCAATTTTTTCTCTTCATATATCAGGAATTTCCTCAATTATAGGA
TCATTAAATTTAATAGTTACAATTATAATAATAAAAAATTTTTCTATAAATTATGACCAAATT
>Seq45 [organism=Apis mellifera mellifera] Apis mellifera mellifera voucher VSU_45, cytochrome oxidase subunit 1 (COI) gene, partial cds; mitochondrial
ATTATTCGAATAGAATTAAGATCCCCAGGATCATGAATTAACAATGATCAAATTTATAATAC
AATTGTTACTAGTCATGCATTCCTAATAATTTTTTTTATAGTTATACCATTTTTAATTGGAGG
ATTTGGAAATTGGCTTATTCCTTTAATACTAGGATCACCTGATATAGCATTCCCCCGAATAA
ATAATATTAGATTTTGATTACTTCCTCCCTCATTATTTATACTTTTATTAAGAAATTTATTTTA
TCCAAGACCAGGAACTGGATGAACAGTATATCCACCATTATCAGCATATTTATATCATTCTT
CACCTTCAGTAGATTTTGCAATTTTTTCTCTTCATATATCAGGAATTTCATCAATTATAGGAT
CATTAAACTTAATAGTTACAATTATAATAATAAAAAATTTTTCTATAAATTATGACCAAATT
>Seq46 [organism=Apis mellifera mellifera] Apis mellifera mellifera voucher VSU_46, cytochrome oxidase subunit 1 (COI) gene, partial cds; mitochondrial
ATTATTAGAATAGAATTAAGATCCCCAGGATCATGAATTAACAATGATCAAATTTATAATAC
AATTGTTACTAGTCATGCATTCCTAATAATTTTTTTTATAGTTATACCATTTTTAATTGGAGG
ATTTGGAAATTGGCTTATTCCTTTAATACTAGGATCACCTGATATAGCATTCCCCCGAATAA
ATAATATTAGATTTTGATTACTTCCTCCCTCATTATTTATACTTTTATTAAGAAATTTATTTTA
TCCAAGACCAGGAACTGGATGAACAGTATATCCACCATTATCAGCATATTTATATCATTCTT
CACCTTCAGTAGATTTTGCAATTTTTTCTCTTCATATATCAGGAATTTCATCAATTATAGGAT
CATTAAACTTAATAGTTACTTTTATAATAATAAAAAATTTTTCTATAAATTATGACCAAATT

>Seq47 [organism=Apis mellifera mellifera] Apis mellifera mellifera voucher VSU_47, cytochrome oxidase subunit 1 (COI) gene, partial cds; mitochondrial
ATTATTCGAATAGAATTAAGATCCCCAGGATCATGAATTAACAATGATCAAATTTATAAT
ACAATTGTTACTAGTCATGCATTCCTAATAATTTTTTTTATAGTTATACCATTTTTAATTG
GAGGATTTGGAAATTGGCTTATTCCTTTAATACTAGGATCACCTGATATAGCATTCCCC
CGAATAAATAATATTAGATTTTGATTACTTCCTCCCTCATTATTTATACTTTTATTAAGAA
ATTTATTTTATCCAAGACCAGGAACTGGATGAACAGTATATCCACCATTATCAGCATAT
TTATATCATTCTTCACCTTCAGTAGATTTTGCAATTTTTTCTCTTCATATATCAGGAATTT
CATCAATTATAGGATCATTAAACTTAATAGTTACAATTATAATAATAAAAAATTTTTCTAT
AAATTATGACCAAATT
>Seq48 [organism=Apis mellifera mellifera] Apis mellifera mellifera voucher VSU_48, cytochrome oxidase subunit 1 (COI) gene, partial cds; mitochondrial
ATTATTCGAATAGAATTAAGATCCCCAGGATCATGAATTAACAATGATCAAATTTATAAT
ACAATTGTTACTAGTCATGCATTCCTAATAATTTTTTTTATAGTTATACCATTTTTAATTG
GAGGATTTGGAAATTGGCTTATTCCTTTAATACTAGGATCACCTGATATAGCATTCCCC
CGAATAAATAATATTAGATTTTGATTACTTCCTCCCTCATTATTTATACTTTTATTAAGAA
ATTTATTTTATCCAAGACCAGGAACTGGATGAACAGTATATCCACCATTATCAGCATAT
TTATATCATTCTTCACCTTCAGTAGATTTTGCAATTTTTTCTCTTCATATATCAGGAATTT
CATCAATTATAGGATCATTAAACTTAATAGTTACAATTATAATAATAAAAAATTTTTCTAT
AAATTATGACCAAATT
>Seq49 [organism=Apis mellifera mellifera] Apis mellifera mellifera voucher VSU_49, cytochrome oxidase subunit 1 (COI) gene, partial cds; mitochondrial
ATTATTCGAATAGAATTAAGATCCCCAGGATCATGAATTAACAATGATCAAATTTATAAT
ACAATTGTTACTAGTCATGCATTCCTAATAATTTTTTTTATAGTTATACCATTTTTAATTG
GAGGATTTGGAAATTGGCTTATTCCTTTAATACTAGGATCACCTGATATAGCATTCCCC
CGAATAAATAATATTAGATTTTGATTACTTCCTCCCTCATTATTTATACTTTTATTAAGAA
ATTTATTTTATCCAAGACCAGGAACTGGATGAACAGTATATCCACCATTATCAGCATAT
TTATATCATTCTTCACCTTCAGTAGATTTTGCAATTTTTTCTCTTCATATATCAGGAATTT
CATCAATTATAGGATCATTAAACTTAATAGTTACAATTATAATAATAAAAAATTTTTCTAT
AAATTATGACCAAATT
>Seq50 [organism=Apis mellifera mellifera] Apis mellifera mellifera voucher VSU_50, cytochrome oxidase subunit 1 (COI) gene, partial cds; mitochondrial
ATTATTCGAATAGAATTAAGATCCCCAGGATCATGAATTAACAATGATCAAATTTATAAT
ACAATTGTTACTAGTCATGCATTCCTAATAATTTTTTTTATAGTTATACCATTTTTAATTG
GAGGATTTGGAAATTGGCTTATTCCTTTAATACTAGGATCACCTGATATAGCATTCCCC
CGAATAAATAATATTAGATTTTGATTACTTCCTCCCTCATTATTTATACTTTTATTAAGAA
ATTTATTTTATCCAAGACCAGGAACTGGATGAACAGTATATCCACCATTATCAGCATAT
TTATATCATTCTTCACCTTCAGTAGATTTTGCAATTTTTTCTCTTCATATATCAGGAATTT

CATCAATTATAGGATCATTAAACTTAATAGTTACAATTATAATAATAAAAAATTTTTCTATAAATTATGACCAAATT

© 2018 by the authors. Submitted for possible open access publication under the terms and conditions of the Creative Commons Attribution (CC BY) license (http://creativecommons.org/licenses/by/4.0/).
